# Analysis of Runoff Variation Characteristics and Influencing Factors in the Wujiang River Basin in the Past 30 Years

**DOI:** 10.3390/ijerph19010372

**Published:** 2021-12-30

**Authors:** Wenxian Guo, Jianwen Hu, Hongxiang Wang

**Affiliations:** School of Water Conservancy, North China University of Water Resources and Electric Power, Zhengzhou 450045, China; guowenxian@ncwu.edu.cn (W.G.); 201602623@stu.ncwu.edu.cn (J.H.)

**Keywords:** Budyko hypothesis, runoff change, climate change, underlying surface, Indicators of Hydrologic Alteration, Wujiang River Basin

## Abstract

Changes in climate and the underlying surface are the main factors affecting runoff. Quantitative assessment of runoff characteristics, and determination of the climate and underlying surface contribution to changes in runoff are critical to water resources management and protection. Based on the runoff data from the Wulong Hydrological Station, combined with the Mann-Kendall test, Indicators of Hydrologic Alteration (IHA), Budyko hypothesis, and changes in climate and the underlying surface, this study comprehensively analyzed the runoff in the Wujiang River Basin (WRB). The results showed that: (1) The annual runoff of Wujiang River showed a downward trend, and an abrupt change occurred in 2005. (2) The overall hydrological change in WRB is 46%, reaching a moderate change. (3) The contribution rates of precipitation (P), potential evaporation (*ET*_0_), and underlying surface to runoff changes are 61.5%, 11.4%, and 26.9%, respectively. (4) After 2005, the WRB has become more arid, human activities have become more active, vegetation coverage has increased, and the built-up land has increased significantly.

## 1. Introduction

Abrupt changes in river runoff will change the habitat conditions to varying degrees, and break the balance of the ecosystem [1]. Therefore, changes in the runoff process have attracted much attention as an essential factor affecting the aquatic ecological environment. The Wujiang River is the largest tributary on the right bank of the upper reaches of the Yangtze River, and one of the 13 largest hydropower energy bases in China. In the context of climate change, coupled with the large-scale cascade hydropower development in the WRB, human intervention in the natural environment of the basin has gradually increased [2]. All have changed the temporal and spatial patterns of the hydrological cycle, and affected the distribution and utilization of regional water resources.

The change of runoff and the quantification of its attributes have always been of concern, and widely studied. Richter et al. [3,4] constructed the Indicators of Hydrologic Alteration (IHA) method to evaluate hydrological change, which is widely used to study river hydrological change and ecological effects. Huang et al. [5] used IHA to assess the hydrological changes and aquatic ecology of the Yangtze River in the past 58 years. Yang et al. [6] used IHA to evaluate the impact of hydrological changes in the middle and lower reaches of the Han River on river fish. Gao et al. [7] evaluated the mechanism change of Yangtze River flow with IHA. Bin Ashraf et al. [8] used IHA to assess the impact of climate change and river regulation on runoff in cold climates. There are two main methods for runoff attribute analysis: a process-based hydrological model, and a statistical method. Different methods have their advantages in quantifying the contribution rate of influencing factors of runoff change [9]. The results can reflect the causes of runoff change to a certain extent, but there are many deficiencies. The former has good physical significance, but requires high-precision data and calibration. Compared with traditional statistical methods, the Budyko hypothesis has better physical significance. Xu et al. [10] analyzed the runoff change in Haihe River Basin by the Budyko framework. Liu et al. [11], based on the Budyko hypothesis, quantified the impacts of climate change and human activities on runoff in the Lancang River Basin. He et al. [12] analyzed the impacts of different weather conditions and land-use change on runoff variations in the Beiluo River Watershed by the Budyko framework.

Wujiang River is an important tributary of the Yangtze River. It is located in the mountainous area of Southwest China. It is one of the major karst areas in the world. In recent years, with the gradual activity of human activities in the WRB, runoff and ecology in the WRB have attracted more and more attention of scholars [13,14]. Zhao et al. [15] analyzed the influence of damming on the distribution and methylation of Hg within a river–reservoir ecosystem in WRB. Wang et al. [16] analyzed the temporal and spatial changes of the flood season in WRB. Wang et al. [17] analyzed the flood change during the past 50 years in WRB.

This article combines IHA, Budyko hypothesis, and underlying surface analysis to establish a new comprehensive analysis framework for runoff changes, and at the same time, fills up the research gap of WRB. The objectives of this study are: (1) to test the variation trend and sudden change year of runoff, rainfall, and potential evaporation; (2) to calculate the hydrological change degree; (3) to analyze the contribution of climate and underlying surface changes to runoff; (4) to analyze the change of drought in the basin; (5) to evaluate the changes of the underlying surface of the basin. This study provides strong theoretical support for the protection and utilization of water resources in the WRB.

## 2. Materials and Methods

### 2.1. Study Area and Dataset

**Figure 1:**
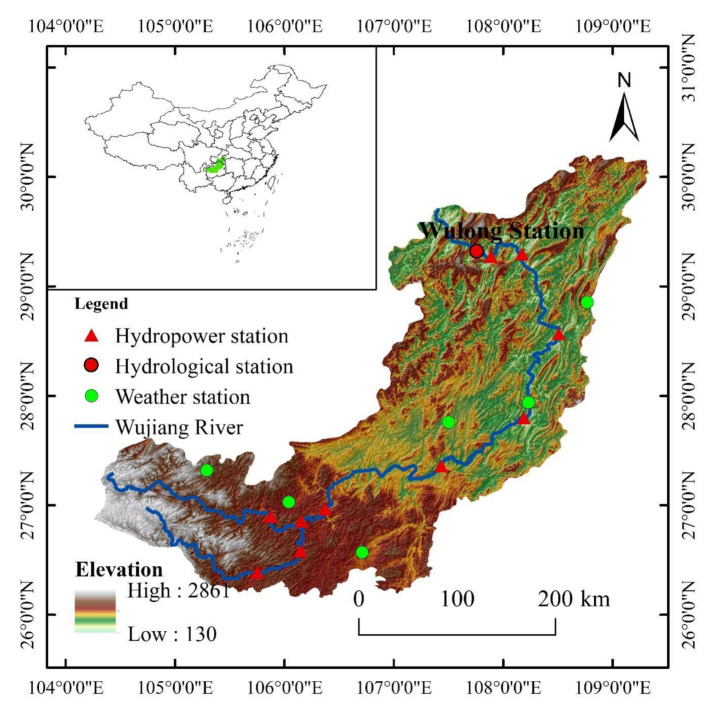
Location of the Wujiang River Basin.

The Wujiang River is the largest tributary on the south bank of the upper reaches of the Yangtze River. It is located between 26°~30° N and 104°~110° E. The drainage area is about 87,900 km^2^, the mainstream is 1037 km in length, and the natural drop is 2124 m. The average annual runoff in the drainage basin is 4.82 × 1010 m^3^. It can be seen from Figure 1 that the Wujiang river system is plume-shaped. The terrain of the basin is high in the southwest, and low in the northeast. Plateaus and hills dominate the terrain. Due to the significant height difference and intense cutting, the abrupt changes of the natural landscape are apparent. With the development of the West-East Power Transmission Project, 10 large and medium-sized hydropower stations have been built in the WRB, making it one of China′s 13 largest hydropower energy bases. In this study, daily runoff data (1990–2019) were obtained from the Hydrological Yearbook of the Yangtze River Basin, and meteorological data (1990–2019) were obtained from the National Meteorological Information Centre (Available online: http://data.cma.cn (accessed on 20 December 2021)). Moreover, the land-use data (1990–2015) and NDVI data (1998–2019) were obtained from the Data Center for Resources and Environmental Sciences of the Chinese Academy of Sciences (Available online: http://www.resdc.cn (accessed on 20 December 2021)). The potential evapotranspiration ($ET_{0}$) was calculated using the Penman–Monteith method.

### 2.2. Methods

#### 2.2.1. Mann-Kendall Test

The Mann-Kendall trend test is a widely used trend non-parametric test [18], with statistics S expressed as the following:

(1) $$S=\sum_{i=1}^{n-1}\sum_{j=i+1}^{n}sgn(x_{j}-x_{i})$$

(2) $$sgn\theta =\left\{\begin{array}{c} 1\\ 0\\ -1 \end{array}\right.\begin{array}{c} ,\\ ,\\ , \end{array}\begin{array}{c} \theta >0\\ \theta =0\\ \theta <0 \end{array}$$

In the form: $x_{i}$,$x_{j}$ represented as a sample data value, n represented as the length of the data collection.

Its test statistic Z is:

(3) $$Z_{c}=\left\{\begin{array}{c} \frac{S-1}{\sqrt{\mathrm{var}(S)}},S>0\\ 0,\;S=0\\ \frac{S+1}{\sqrt{\mathrm{var}(S)}},S<0 \end{array}\right.$$

(4) $$\mathrm{var}(S)=[n(n-1)(2n+5)-\sum_{t}t(t-1)(2t+5)]/18$$

In this trend test, Z>0 represents an increasing trend, whereas Z<0 represents a decreasing trend. $\left|Z\right|>1.96$ and $\left|Z\right|>2.56$ indicate significance levels of 95% and 99%, respectively. The MK test was used to test the significance of annual meteorological and hydrological trends in the study area. At the same time, the same statistical calculation is made for the reverse sequence of the original time series, so that, if the two curves appear at the intersection within the 95% confidence level, it indicates a mutation at that point in time.

#### 2.2.2. Budyko Hypothesis

The Budyko hypothesis considers the impact of potential evaporation on runoff change compared with the traditional mathematical–statistical method, and its physical significance is clearer, which is often used in the attribution identification of runoff change.

The long-term water balance of the basin can be expressed as:

(5) R=P−E−ΔS

where R is the average runoff depth (mm); P is the average precipitation (mm); E is the average actual evaporation (mm); ΔS is the change of water storage (mm). In the analysis of long-time scale runoff change, ΔS is generally assumed to be 0.

Choudhury and Yang [19,20] used dimensional analysis and quantitative statistics to deduce the water–energy balance equation on an annual average scale, based on the Budyko hypothesis, and paired with the empirical formula of yearly evaporation. The following is the expression:

(6) $$E=\frac{P\times ET_{0}}{{\left(P^{n}+ET_{0}^{n}\right)}^{\frac{1}{n}}}$$

where *n* is the underlying surface parameter.

Combined with Equations (5) and (6), the water balance equation can be expressed as the following formula:

(7) $$R=P-\frac{P\times ET_{0}}{{\left(P^{n}+ET_{0}^{n}\right)}^{\frac{1}{n}}}$$

The following completely differential form can be used to depict the variance in yearly runoff depth R:

(8) $$dR=\frac{\partial R}{\partial P}dP+\frac{\partial R}{\partial ET_{0}}dET_{0}+\frac{\partial R}{\partial n}dn$$

Combined with the water balance equation, the elastic coefficient of runoff on each influencing factor can be expressed as follows:

(9) $$\varepsilon_{x}=\frac{\partial R}{\partial x}\times \frac{x}{R}$$

According to the definition of these elastic coefficients, divide Equation (8) by multi-year average runoff depth R to obtain:

(10) $$dR=\frac{\partial R}{\partial P}dP+\frac{\partial R}{\partial ET_{0}}dET_{0}+\frac{\partial R}{\partial n}dn$$

According to the analysis of mutation results, the runoff depth in the base period is recorded as $R_{1}$, the runoff depth in the mutation period is $R_{2}$, and the difference of runoff depth between the two periods is ΔR.

(11) $$\Delta R=R_{2}-R_{1}$$

According to the elastic coefficient of runoff on each influencing factor, the change caused by the corresponding factor on the runoff depth can be calculated, expressed as $\Delta R_{x}$; x represents P, $ET_{0}$, or n.

(12) $$\Delta R_{x}=\varepsilon_{x}\frac{R}{x}\Delta x$$

The calculated runoff depth variation ΔR′ is obtained by summing, which is expressed as follows:

(13) $$\Delta R\prime =\Delta R_{P}+\Delta R_{ET_{0}}+\Delta R_{n}$$

The contribution rate of each factor to the change of runoff is calculated according to the following formula:

(14) $$\eta_{P}=\frac{\Delta R_{P}}{\Delta R\prime }\times 100\%\;\eta_{ET_{0}}=\frac{\Delta R_{ET_{0}}}{\Delta R\prime }\times 100\%\;\eta_{n}=\frac{\Delta R_{n}}{\Delta R\prime }\times 100\%$$

#### 2.2.3. Indicators of Hydrologic Alteration

Indicators of Hydrologic Alteration (IHA) is widely used to evaluate the change degree of the river hydrological situation. The Wulong Hydrological Station′s daily discharge data is counted, and long-term hydrological data in the natural condition is utilized to define the range of hydrological variables. Thirty-two hydrological indicators are divided into five groups (Table 1) based on basic hydrological regime characteristics (this study excludes the hydrological indicator of zero flow days), and 25% and 75% of the occurrence probability of each indicator are taken as the upper and lower limits of IHA, i.e., IHA threshold. Then, the degree of change before and after the abrupt change of hydrological regime is analyzed to determine the changes in natural rivers caused by human activities. The formula is as follows:

(15) $$D_{m}=\left|\frac{N_{m}-N_{\varepsilon }}{N_{\varepsilon }}\right|\times 100\%$$

(16) $$D_{0}=\sqrt{\frac{1}{N}\sum_{i=1}^{N}D_{i}^{2}}$$

where $D_{m}$ is the change degree of the *m*-th index; $N_{m}$ is the number of observation years that the *m*-th index falls within the IHA threshold after change; $N_{\varepsilon }$ is the number of years expected to fall within the IHA threshold after the change of the *m*-th index; N is the number of hydrological indicators; $D_{0}$ is the change of overall hydrological characteristics.

When 0 < $D_{m}$ and $D_{0}$ ≤ 33%, there is no change or low change; when 33% < $D_{m}$ and $D_{0}$ ≤ 67%, it is a moderate change; when 67% < $D_{m}$ and $D_{0}$ ≤ 100%, it is a high level of change.

## 3. Result

### 3.1. Trend and Mutation Analysis

**Figure 2:**
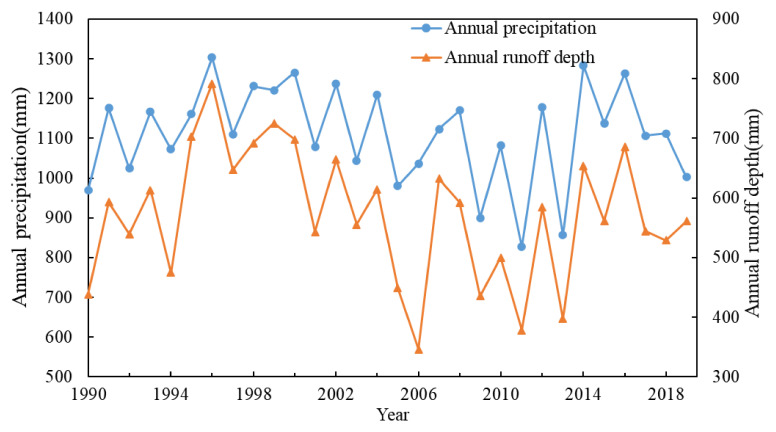
Precipitation and runoff depth inter-annual variation trends in the Wujiang River Basin from 1990 to 2019.

**Figure 3:**
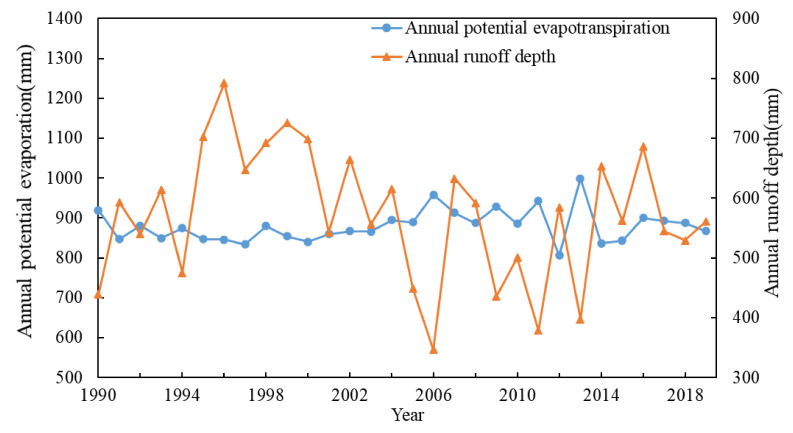
Potential evaporation and runoff depth inter-annual variation trends in the Wujiang River Basin from 1990 to 2019.

Figure 2 and Figure 3 are the process charts of annual runoff depth, precipitation, and potential evaporation in WRB from 1990 to 2019. The Mann-Kendall trend and mutation test results for runoff depth (R), precipitation (P), and potential evaporation (*ET*_0_) are shown in Table 2. The runoff depth and precipitation test statistics are −1.249 and −0.357, less than 1.96, which fail to pass the 95% significance level test, indicating the overall average annual runoff depth and average annual precipitation of Wujiang River show a downward trend, but it is not significant. The test value of potential evaporation is 1.463, less than 1.96, indicating that the potential evaporation in WRB shows an upward trend, but it is not significant.

The abrupt years of runoff depth are 2005, 2015, and 2018; the abrupt years of rainfall are 2005, 2015, and 2017; and the abrupt change of potential evaporation occurred in 2004. The year 2005 is selected as the abrupt year of runoff.

### 3.2. IHA Hydrological Regime Analysis

To assess the degree of hydrological change in the runoff of the WRB, with 2005 as the demarcation point, the daily flow data of the Wulong Hydrological Station over the years were divided into two research periods: 1990–2004 is the study of the base flow sequence under the natural state before the hydrological abrupt change period; and 2005–2019 is the stage of change. The results are shown in Table 3:

#### 3.2.1. Change of Monthly Median Flow

**Figure 4:**
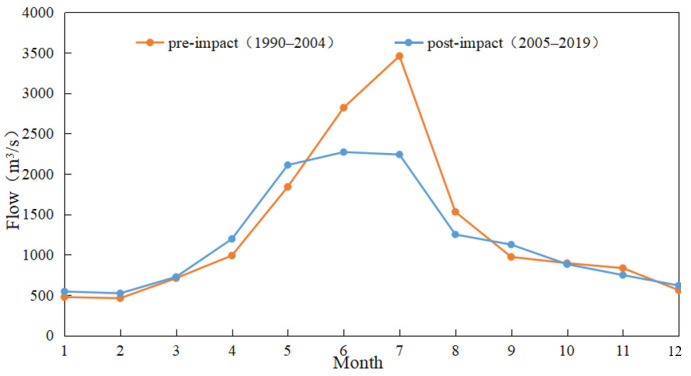
Comparison of monthly median flow of Wulong Station in the different periods.

Figure 4 shows the difference between the median monthly discharge at the Wulong Hydrological Station around 2005. As can be seen from the figure, since the abrupt change in 2005, the median monthly flow of the Wulong Hydrological Station has shown a downward trend as a whole, with the most significant decline in flow in the three months from June to August, and a slight increase in flow in January, February, April, and May. The overall change of the median monthly flow of the Wulong Hydrological Station is 50%, which is a moderate change. Analysis of the reasons: 1. rainfall in the WRB decreases during the flood season; 2. reservoir storage in the flood season leads to flow reduction.

#### 3.2.2. Change of Annual Extreme Flow

**Figure 5:**
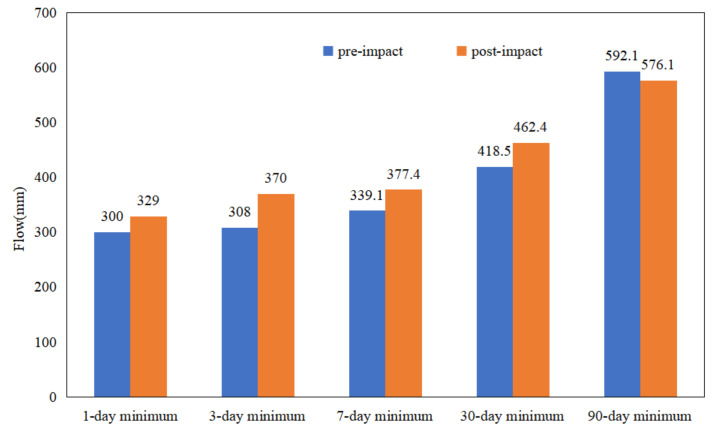
Comparison of minimum flow in the different periods.

**Figure 6:**
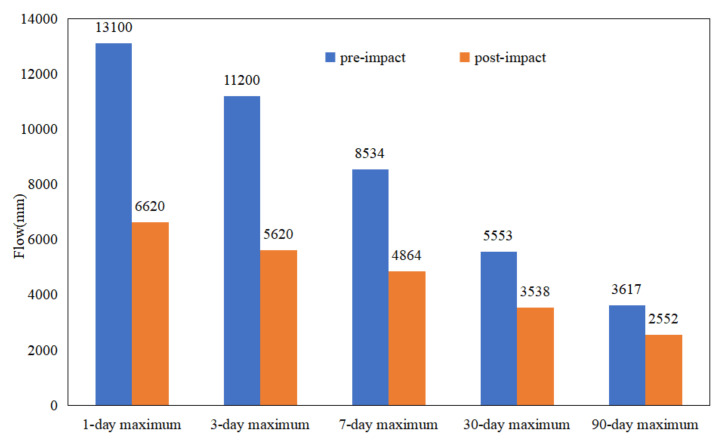
Comparison of maximum flow in the different periods.

Table 1, and Figure 5 and Figure 6 demonstrate that the annual minimum flow of Wulong Station in 1-d, 3-d, 7-d, and 30-d increased due to hydrologic alteration. The annual maximum flow decreased to a certain extent at 1-d, 3-d, 7-d, 30-d, and 90-d. At the same time, the base flow index of WRB also increased from 0.2199 to 0.2847. The above results are consistent with the results caused by the reduction of annual rainfall in the sudden change period, and the “flood storage” of the reservoir [21,22].

#### 3.2.3. Change in the Occurrence Time of Annual Extreme Flow

The change degrees of occurrence time of annual minimum and annual maximum flow at Wulong Hydrological Station are 40% and 20%, respectively. The minimum flow was delayed by 25 days, whereas the maximum flow was advanced by 4 days.

#### 3.2.4. Change of Flow Pulse and Flow Change Rate

After the hydrological change, the Wulong Station′s high pulses count and duration decreased to a certain extent. The number of high pulses reduced from 11 to 10, and the duration reduced from 4 d to 3.5 d. The number of low pulses increased from 6 to 15, and the duration reduced from 6 d to 2 d. Among the changes, the frequency change of low pluses count is the most obvious, and the change degree reaches 66.66%. Analysis reason: the construction of the reservoir in the upper reaches of Wujiang River reduces the frequency and duration of high pulses to a certain extent, and increases the number of low pulses [23,24].

#### 3.2.5. Overall Change Degree of Hydrology

**Figure 7:**
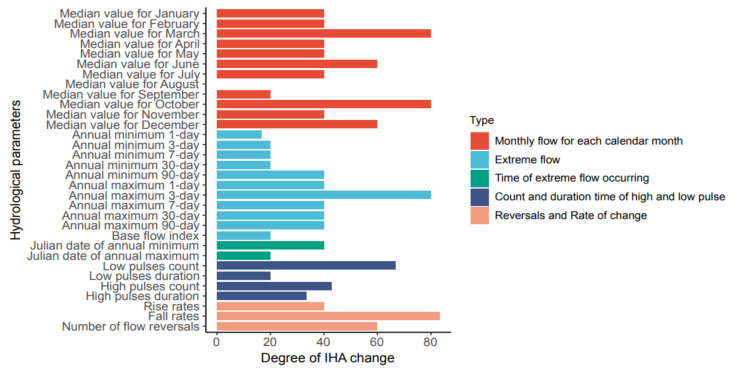
Degrees of Indicators of Hydrologic Alteration in the Wujiang River Basin.

In order to explore the influence of WRB hydrological change on water system hydrological condition, the absolute values of 32 hydrological indexes before and after the change of Wulong Hydrological Station are calculated. The results are shown in Figure 7. According to the information in the figure, most hydrological indicators of Wulong Hydrological Station are in the area of moderate change. There are four hydrological indicators (median value for March, median value for October, 3-day maximum, fall rate) with high levels of change, accounting for 12.5% of the total hydrological indicators, of which the variation of fall rate is the most obvious, and the degree of hydrological variation is 83%; there are nine hydrological indicators (median value for August, median value for September, 1-day minimum, 3-day minimum, 7-day minimum, 30-day minimum, base flow index, date of maximum, low pulse duration) with low change, accounting for 28% of the total hydrological indicators, of which the change degree of median flow in August is the lowest, and its hydrological degree of change is 0. Calculations were performed of the change degree of hydrological indicators of each group, and the results are shown in Table 4. It can be seen from the results in the table that, except for the third group of indicators with a low change degree, all other indicators of Wulong Hydrological Station belong to a moderate change degree; the change degree of the fifth group was 64%, close to a change of a high level; the change degree of overall hydrological index is 46.17%, which belongs to a moderate change [25].

### 3.3. Attribution Analysis

#### Attribution Analysis of Runoff Change

Table 5 illustrates the elastic coefficients of climatic and underlying surface parameters at the WL station in WRB before and after the abrupt runoff. The elastic coefficients of runoff depth to precipitation, potential evapotranspiration, and underlying surface are 1.54, −0.54, and −0.55, respectively, from the perspective of the entire study period, suggesting that runoff is negatively correlated with *ET*_0_ and *n*, but positively correlated with P. It also demonstrates that when precipitation rises by 1%, the runoff depth increases by 1.54%, whereas a potential evapotranspiration rise by 1% decreases runoff depth by 0.544%. The absolute values of the elastic coefficients of $\varepsilon_{P}$, $\varepsilon_{ET_{0}}$, $\varepsilon_{n}$, and *n* are increased compared to the base period, indicating that the WRB runoff is more sensitive to changes in the environment and the underlying surface during the abrupt period.

Based on Equations (11)–(13), the specific results of $\Delta R_{P}$, $\Delta R_{ET_{0}}$, $\Delta R_{n}$, ΔR′, and ΔR are shown in Table 6. The difference between the calculated runoff depth change (ΔR′ = −96.06 mm) and the actual runoff depth change (ΔR = −96.2 mm) is slight, implying that the methodology and findings used in this study are adequate for assessing the impact of changes in climate and the underlying surface on runoff. Compared to the base period, annual runoff depth fell by 96.19 mm, precipitation decreased by 74.71 mm, potential evaporation rose by 31.89 mm, and the underlying surface coefficient increased by 0.096 mm. Rainfall had the greatest impact, reducing runoff depth by 59.03 mm, accounting for 61.45%; the underlying surface came in second, reducing runoff depth by 25.84 mm, accounting for 26.9%; and potential evapotranspiration had the least impact, resulting in a reduction of runoff depth by 11.19 mm, accounting for 11.65%. Precipitation is the biggest contributor to runoff change in WRB.

## 4. Discussion

### 4.1. The Impact of Climatic Change

**Figure 8:**
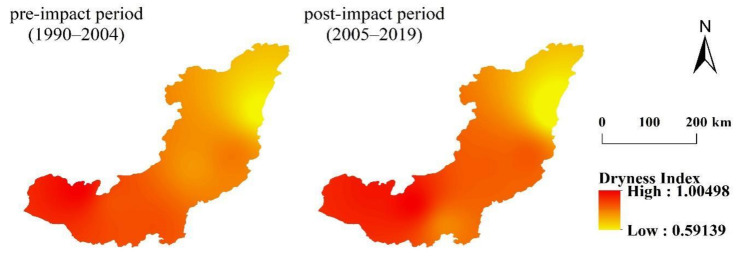
Spatial distribution of dryness index in Wujiang River Basin in the difference period.

According to Table 6, the runoff reduction in WRB is mainly caused by climate change. According to the daily meteorological data of six meteorological stations in WRB, the annual dryness index (*ET*_0_/P) in the base period and change period is determined, and the distribution of the dryness index in the study area is obtained, by inverse distance interpolation [26]. The results are shown in Figure 8: in the base period, the maximum dryness index is 0.9624, the minimum is 0.5913, and the average is 0.79611. The distribution characteristics show a decreasing trend from southwest to northeast. The upper reaches of the Wujiang River are relatively arid, and the lower reaches have the smallest dryness index, and are relatively humid. The minimum dryness index in the change period is 0.6502, the maximum is 1.005, and the mean value is 0.8783. The distribution characteristics still show a decreasing trend from southwest to northeast. However, compared with the base period, the middle reaches of WRB in the change period become more arid. The average value of the overall dryness index of the basin also increases relatively, indicating that the WRB becomes more arid in the change period.

Annual runoff depth fell by 96.19 mm, precipitation decreased by 74.71 mm, potential evaporation rose by 31.89 mm, and the underlying surface coefficient increased by 0.096 mm.

### 4.2. The Impact of Underlying Surface Change

The underlying surface parameter *n* is mainly related to the basin′s topography, soil, land-use, vegetation, and reservoir [27,28]. It is generally believed that the terrain and soil are relatively stable, and change little in a short time. Therefore, the value of *n* is mainly related to land-use and vegetation factors, and the construction of reservoirs. In order to further explore the main influencing factors of parameter *n*, statistical analysis was carried out on land-use, vegetation cover changes, and the cumulative reservoir capacity of the watershed [29].

#### 4.2.1. Land-Use

**Figure 9:**
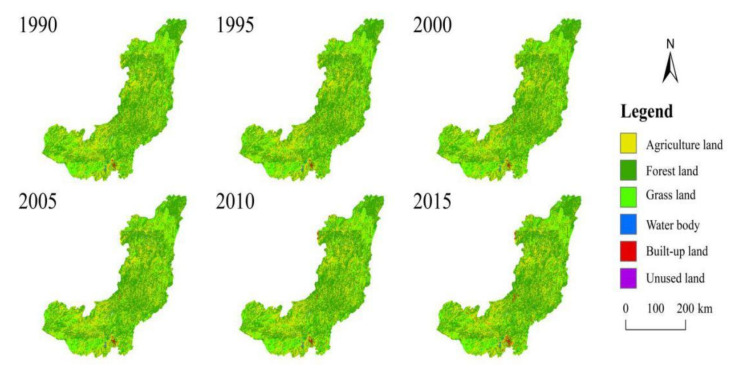
Spatial distribution of land-use in Wujiang River Basin in 1990, 1995, 2000, 2005, 2010, 2015.

Figure 9 and Table 7 show the land-use and land distribution in the study area from 1990 to 2015. The land-use types in the WRB are mainly forest land, accounting for 51~52% [30]. The land-use types are from large to small: forest land > agriculture land > grass land > built-up land > water body > barren land [31]. Except for the significant increase in built-up land, other land types have not changed much. The built-up land areas increased from 300.9 to 958.9 km^2^. Significantly since 2005, the area of built-up land has increased from 340.68 to 958.92 km^2^. This shows that human activities have become more active after 2005, the same as the abrupt year of runoff. The increase of human activities and built-up land is also the reason for the underlying surface parameter *n* change [32].

#### 4.2.2. Vegetation Cover

**Figure 10:**
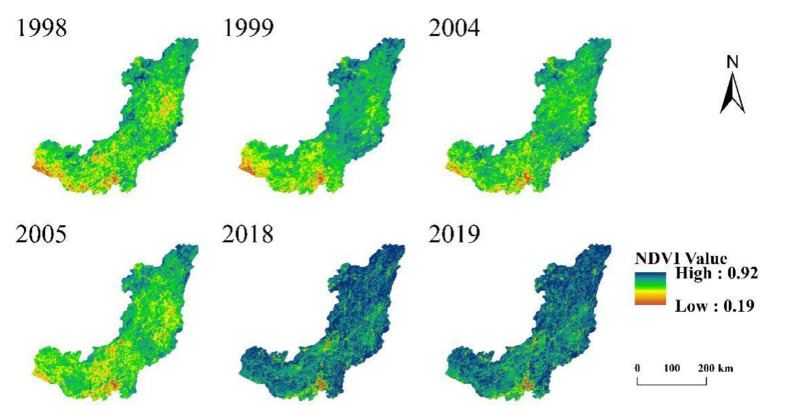
Spatial distribution of NDVI in Wujiang River Basin in 1998, 1999, 2004, 2005, 2018, 2019.

**Figure 11:**
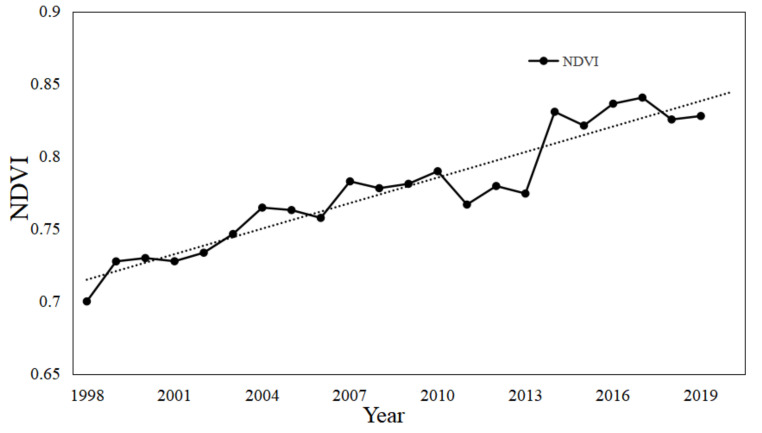
NDVI change at the Wulong Station during the period 1998–2019.

From the Hydrothermal Coupling equilibrium equation (Equation (6)), it can be seen that under the same conditions, when the value of parameter *n* is large, *ET*_0_ is larger [33,34]. It is generally believed that afforestation and vegetation improvement will lead to greater water consumption, and vegetation coverage is positively correlated with potential evaporation. A large number of studies also show that there is a close relationship between vegetation and parameter *n* [35]. Under other similar conditions, the parameter *n* of the watershed with broad vegetation coverage is usually greater than that of the watershed with small vegetation coverage. In order to reveal the change of vegetation over time, normalized vegetation index (NDVI) is used to describe the change of vegetation cover. Figure 10 shows the vegetation cover map of typical years in the study area. Figure 11 shows the interannual variation trend of NDVI in the study area from 1998 to 2019.As can be seen from Figure 10 and Figure 11, the NDVI of WRB showed an upward trend from 1998 to 2019. The project of returning farmland to the forest in WRB may be the main reason for the increase of NDVI in the basin, and may also be one of the reasons for the increase of the underlying surface parameter *n* in the abrupt period.

#### 4.2.3. Construction of Reservoirs

**Figure 12:**
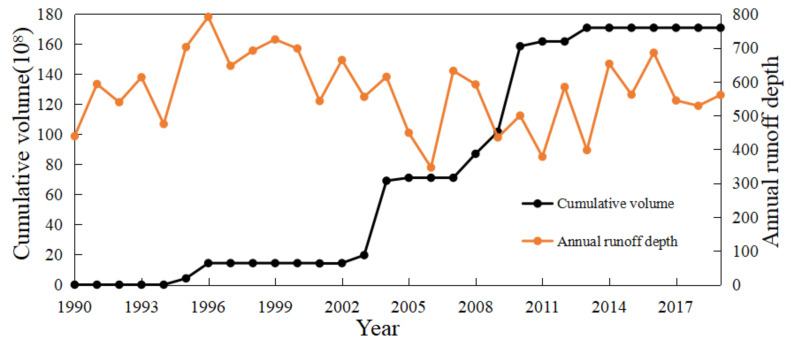
Variation of cumulative reservoir volume in the WRB.

As we all know, the construction of reservoirs impacts the change of river runoff [36,37,38]. Wang et al. [39] conducted an attribution analysis on the runoff of 413 watersheds in the United States. The results show that in most non-urban watersheds, the direct impact of humans on the average annual runoff can be attributed to several human activities, such as farmland expansion, irrigation, and reservoir construction. The construction of reservoirs increases human water consumption (e.g., water supply, hydropower supply, and irrigation) and water loss caused by an evaporation enhancement of the large surface area of the water storage body. Figure 12 shows the variation of cumulative reservoir volume; 2003–2010 are the concentrated years for the construction of Wujiang reservoir, during which, six large hydropower stations were started. These are all manifestations of human activities becoming more active in the WRB.

## 5. Conclusions

During the period 1990–2019, this study used a comprehensive framework to evaluate the many characteristics and attribution of runoff change in WRB. The following is a summary of the main conclusions:

1. Yearly runoff depth and precipitation in the WRB exhibited a slight downward trend from 1990 to 2019, with an abrupt change in 2005. Potential evapotranspiration shows a tiny upward trend.
2. Through the analysis of Wujiang River before and after 2005, among the 32 IHA hydrological change degree indicators selected, 4 indicators are a high level of change, 19 indicators are a moderate change, and 9 indicators are a low change; the comprehensive hydrological change degree of the basin is 46%; and the overall flow is reduced, which belongs to a moderate change.
3. Based on the Budyko Framework and Choudhury–Yang equation (Equation (6)), the contribution of climate change and the change of the underlying surface to runoff reduction is 73.1% and 26.9%, respectively. The dry climate in the basin is the main reason for the decrease in runoff depth. The decrease in runoff is also due to an increase in the underlying surface parameter *n*. After 2005, the WRB′s vegetation coverage rose, the extent of built-up land expanded, and reservoir development increased. These are the factors that have contributed to the decrease in the runoff.

## Figures and Tables

**Table 1:** Summary of hydrologic parameters used in the IHA.

IHA Statistics Group	Regime Characteristics	Hydrologic Parameters
Group 1: Magnitude of monthly flow conditions	Magnitude and timing	Monthly flow for each calendar month
Group 2: Magnitude and duration of annual extreme flow conditions	Magnitude and duration	Annual minimum 1-day, 3-day, 7-day, 30-day, and 90-day medians Annual maximum 1-day, 3-day, 7-day, 30-day, and 90-day medians
Group 3: Timing of annual extreme flow conditions	Timing	Date of annual 1-day maximum flowDate of annual 1-day minimum flow
Group 4: Frequency and duration of high and low pulses	Magnitude, frequency, and duration	Number of high pulses in each year Number of low pulses in each year Median duration of the annual high pulse Median duration of the annual low pulse
Group 5: Rate and frequency of flow condition changes	Frequency and rate of change	Medians of all positive differences between consecutive daily values (rise rate) Medians of all negative differences between consecutive daily values (fall rate) Number of reversals

**Table 2:** Trend and mutation test results of runoff depth and precipitation and potential evaporation in Wujiang.

Paraments	R	P	*ET*_0_
Statistic	−1.249	−0.3568	1.463
Test	$\left|Z_{c}\right|<1.96$	$\left|Z_{c}\right|<1.96$	$\left|Z_{c}\right|<1.96$
Abrupt year	2005, 2015, 2018	2005, 2015, 2017	2004

**Table 3:** Results of IHA change degree under different periods at Wulong Station.

Parameter (i)	Median Value		IHA Threshold		$N_{mi}$	$N_{\varepsilon i}$	$D_{i}/\%$
	Pre-Impact Period	Post-Impact Period	Low	High			
January	475	544	436.2	555.8	3	5	−40
February	461	522	418.8	507.8	3	5	−40
March	709	725	522.1	823.8	9	5	80
April	990	1195	842.7	1086	3	5	−40
May	1840	2110	1686	1989	3	5	−40
June	2820	2270	2138	3237	8	5	60
July	3460	2240	2442	3729	7	5	40
August	1530	1250	1172	2244	5	5	0
September	972.5	1125	891.7	1194	4	5	−20
October	895	882	822.7	1344	9	5	80
November	834	747.5	622.3	858.9	7	5	40
December	561	618	520.2	617.6	2	5	−60
1-day minimum	300	329	280.2	342	7	6	16.67
3-day minimum	308	370	294.8	353.8	6	5	20
7-day minimum	339.1	377.4	323.1	381.4	6	5	20
30-day minimum	418.5	462.4	393.1	443.9	4	5	−20
90-day minimum	592.1	576.1	512.7	636.1	7	5	40
1-day maximum	13,100	6620	11,980	16,260	3	5	−40
3-day maximum	11,200	5620	9433	13,660	1	5	−80
7-day maximum	8534	4864	7756	10,530	3	5	−40
30-day maximum	5553	3538	4615	6121	3	5	−40
90-day maximum	3617	2552	3400	3880	3	5	−40
Base flow index	0.2199	0.2847	0.1949	0.2512	4	5	−20
Date of minimum	32	57	26.96	48.44	3	5	−40
Date of maximum	187	183	178.4	193.4	4	5	−20
Low pulse count	6	15	4.28	8	2	6	−66.66
Low pulse duration	6	2	3.42	8.44	4	5	−20
High pulse count	11	10	10	12.72	4	7	−42.86
High pulse duration	4	3.5	3.64	5	4	6	−33.33
Rise rate	73	100	67.84	88.32	3	5	−40
Fall rate	−67	−115	−80	−63.28	1	6	−83.33
Number of reversals	136	178	130.8	145.9	2	5	−60

**Table 4:** Statistics of degrees of Indicators of Hydrologic Alteration of Wujiang River.

Hydrological Station	Hydrological Change Degree of Each Group					Overall Hydrological Change
	Group 1	Group 2	Group 3	Group 4	Group 5	
Wulong	50% (M)	38% (M)	32% (L)	44% (M)	64% (M)	46% (M)

**Table 5:** Statistics of hydrological and climatic factors in the Wujiang River Basin during the study period.

Period	P/mm	R/mm	*ET*_0_/mm	*n*	Elasticity Coefficients		
					$\varepsilon_{P}$	$\varepsilon_{ET_{0}}$	$\varepsilon_{n}$
1990–2004	1151.45	619.71	863.81	1.123	1.498	−0.4977	−0.52
2005–2019	1076.74	523.52	895.7	1.219	1.587	−0.5878	−0.595
1990–2019	1114.10	571.62	879.76	1.168	1.54	−0.54	−0.55

**Table 6:** Contributions of underlying surface and climatic factors to the changes in runoff during the study period.

$\Delta R_{P}$	$\Delta R_{ET_{0}}$	$\Delta R_{n}$	ΔR′	ΔR	$\eta_{P}$	$\eta_{ET_{0}}$	$\eta_{n}$
−59.03	−11.19	−25.84	−96.06	−96.2	61.45%	11.65%	26.90%

**Table 7:** Change in land-use area in different years in the Wujiang River Basin.

Land-Use Pattern (km^2^) (%)						
Year	Agriculture Land	Forest Land	Grass Land	Water Body	Built-up Land	Barren Land
1990	26,603.3 (30.27%)	45,710.5 (52.00%)	14,974.0 (17.04%)	299.6 (0.34%)	300.6 (0.34%)	12.0 (0.01%)
1995	26,595.3 (30.26%)	45,699.5 (51.99%)	14,990.0 (17.05%)	300.6 (0.34%)	302.6 (0.34%)	12.0 (0.01%)
2000	26,826.7 (30.52%)	45,218.5 (51.44%)	15,216.5 (17.31%)	303.6 (0.35%)	322.6 (0.37%)	12.0 (0.01%)
2005	26,880.8 (30.58%)	45,665.4 (51.95%)	14,685.4 (16.70%)	316.6 (0.36%)	340.7 (0.39%)	11.0 (0.01%)
2010	26,748.6 (30.43%)	45,745.6 (52.04%)	14,642.3 (16.65%)	322.6 (0.37%)	429.9 (0.49%)	11.0 (0.01%)
2015	26,414.9 (30.05%)	45,610.3 (51.89%)	14,564.2 (16.57%)	342.7 (0.39%)	958.9 (1.09%)	9.0 (0.01%)

## Data Availability

Not applicable.
